# Early versus delayed enteral nutrition in mechanically ventilated patients with circulatory shock: statistics fallacy of logistic regression model

**DOI:** 10.1186/s13054-022-04196-6

**Published:** 2022-10-18

**Authors:** Shohei Ono

**Affiliations:** 1 Department of Emergency and Critical Care Medicine, Tokyo Metropolitan Tama Medical Center, 2-8-29 Musashidai, Fuchu-Shi, Tokyo, 183-8524 Japan; 2 Department of Anesthesiology and Critical Care Medicine, Jichi Medical University, Saitama Medical Center, 1-847 Amanuma-Cho, Omiya-Ku, Saitama-Shi, Saitama, 330-8503 Japan

**Dear editor**,

I read the article by Luis Ortiz-Reyes et al. [1] with great interest and appreciate the value of the authors’ efforts to assess the association between the timing of enteral nutrition (early vs. delayed) and mortality in patients with circulatory shock on mechanical ventilation. If this study determines the optimal timing of enteral feeding, we may be able to improve patient outcomes without the risk of intestinal ischemia or other complications. However, we would like to point out three areas of concern regarding the statistical analysis.

First, the authors should note that logistic regression models may not demonstrate a causal relationship. Nonlinear probability models (NLPM), including logistic regression, always perform suboptimally unless all the relevant covariates are included; thus, it is difficult to infer causality based on the results [2]. Although the statistical significance of the results of NLPM may be unaffected by attenuation bias, the authors should demonstrate the average treatment effect if they wish to establish a causal relationship clearly.

Second, the authors use the NUTRIC score, APACHE II score, and age as explanatory variables in their multivariate analysis, but these should not be entered into the model simultaneously. The NUTRIC score includes age and APACHE II, so the explanatory variables are not mutually independent. If the authors select only the appropriate components of the NUTRIC score for the model, they will be able to increase its goodness of fit. Furthermore, when using the NUTRIC score, they should show the variance inflation factor (VIF) to check for multicollinearity [3].

Third, the P value for interaction should not be used for logistic models. When the product term in a linear model is statistically significant, an interaction effect is considered to exist because the slope changes. However, if the model is nonlinear, the interaction effect cannot be evaluated simply by looking at the statistical significance of the coefficient on the interaction term because these are already interactive before the product term is introduced into the model [2, 4, 5]. The authors should show only the P value for the stratified subgroup analyses or marginal effect [4].

## Data Availability

Not applicable.
